# Twenty-seven years of screening for Shiga toxin-producing *Escherichia coli* in a university hospital. Brussels, Belgium, 1987-2014

**DOI:** 10.1371/journal.pone.0199968

**Published:** 2018-07-02

**Authors:** Klara De Rauw, Steve Jacobs, Denis Piérard

**Affiliations:** Vrije Universiteit Brussel (VUB), Universitair Ziekenhuis Brussel (UZ Brussel), Department of Microbiology and Infection Control, Belgian National Reference Centre for STEC/VTEC, Brussels, Belgium; Hospital General O’Horan, MEXICO

## Abstract

**Objective:**

Since 1987 all fecal samples referred to the clinical microbiology laboratory of the UZ Brussel were screened for the presence of Shiga toxin-producing *E*. *coli* (STEC). In this study all STEC strains isolated over a period of 27 years (1987–2014) were reexamined to achieve deeper insight in the STEC infections in our patient population.

**Methods:**

A total of 606 STEC strains from 604 patients were subjected to molecular methods for shiga toxin (*stx*) subtyping, detection of additional virulence genes, typing of the O-serogroups, and phylogenetic relatedness assessment of STEC O157:H7/H-.

**Results:**

Since the introduction of PCR in 1991 the annual positivity rates varied between 1.1% and 2.7%. The isolation rate of STEC O157:H7/H- remained stable over the years while the isolation rate of non-O157 serotypes increased, mainly since 2011. The majority of the patients were children. Uncomplicated- and bloody diarrhea were the most prevalent gastrointestinal manifestations (respectively 51.9% and 13.6%), 4.3% of the strains were related to the hemolytic uremic syndrome (HUS), and 30.2% of the patients showed none of these symptoms. The strains were very diverse; they belonged to 72 different O-serovars and all *stx* subtypes except *stx1d* and *stx2g* were identified. Out of the 23 *stx2f*-positives one was associated with HUS and one belonged to the *E*. *albertii* species. As seen in other studies, the frequency of strains of the O157:H7/H- serotype and strains carrying *stx2a*, *eaeA* and *ehxA* was higher in patients with HUS.

**Conclusions:**

The characteristics and trends of STEC infection seen in our patient population are similar to those noted in other countries. STEC infections in our hospital are mainly sporadic, and a substantial portion of the patients were asymptomatic carriers. Human STEC Stx2f infection was less rare than previously assumed and we report the first Belgian STEC *stx2f* HUS case and *stx2f* positive *E*. *albertii* infection.

## Introduction

Ever since the first description of an outbreak of hemorrhagic colitis (HC) associated with Shiga toxin-producing *Escherichia coli* (STEC) in the United States in 1983, STEC have been identified worldwide as an important cause of human disease, mostly foodborne [1]. In addition of uncomplicated to severe bloody diarrhea, STEC infections can evolve into the development of the life-threatening hemolytic uremic syndrome (HUS) [2]. The World Health Organization (WHO) estimated that STEC infections could account for 1176854 cases of foodborne illness and 128 cases of foodborne deaths in 2010 [3]. According to the European Centre for Disease Prevention and Control (ECDC) the notification rate of STEC infections between 2010 and 2012 was 1.7 cases/100000 [4]. However, these notification data probably underestimate the true incidence of STEC infection and a recent study conducted in Germany estimated the true annual incidence of STEC-associated gastrointestinal infections at 35/100000 [5].

Production of Shiga toxins (Stx) are STEC’s main virulence trait. There are two types of Shiga toxins, Stx1 and Stx2, which are further subdivided in three Stx1 (1a, 1c, 1d) and seven Stx2 (2a-2g) subtypes based on differences in their gene sequences [6]. Besides Stx production, the formation of “attaching and effacing” lesions (A/E-lesions) in the gut mucosa, a characteristic shared with enteropathogenic *E*. *coli* (EPEC), is considered one of the most important virulence properties of STEC. Genes responsible for the A/E phenotype are located on the chromosomal locus for enterocyte effacement (LEE) pathogenicity island and include *eaeA* encoding the outer membrane protein intimin mediating the attachment to the enterocytes [7]. In addition, many STEC strains harbor a high molecular plasmid containing the *ehxA* gene encoding for a hemolysin called enterohemolysin (8). Serotype O157:H7 was considered the main serotype related to STEC infection at first, but over the years a very heterogeneous group of STEC serotypes have been reported to cause human disease. In order to get grip on this heterogeneous group a classification of the STEC serotypes in five "seropathotypes" (A through E) based on their occurrence and association with severe human disease and outbreaks was proposed by Karmali *et al*. in 2003 [8]. Seropathotype A consists solely of the serotype O157:H7/H-, while seropathotypes B–D include non-O157 serotypes classified according to their involvement in outbreaks and/or HUS [8]. However, in the spring of 2011 a very large and severe outbreak of HUS was caused by a rare hybrid Stx-producing enteroaggregative *E*. *coli* (Agg-STEC) of serotype O104:H4 underlining the possible danger of seemingly innocent STEC non-O157 serotypes. These strains carried genes involved in enteroaggregative adherence to enterocytes, including *aaiC* and *aggR*, as well as the *stx* gene [9].

Since 1987 all human fecal samples referred to the laboratory of microbiology of the Universitair Ziekenhuis (UZ) Brussel university hospital of Brussels for aerobic culture have been systematically screened for the presence of STEC O157 as well as non-O157 [10–13]. As a result of its long-standing experience our laboratory was designated as the Belgian National Reference Centre (NRC) for STEC in 2011 and in this function it has been responsible for the surveillance of human STEC infections in Belgium ever since [14].

Because we wanted to gain a broader knowledge of the characteristics of STEC infections in the patient population of UZ Brussel, we reexamined all stored strains isolated from in- and outpatients over a period of 27 years (1987–2014) using the typing methods currently in place at our laboratory.

## Materials and methods

### Historical STEC screening, strain collection and serotyping

All fecal samples, from in- and outpatients, submitted directly for bacterial culture to the clinical laboratory of microbiology of the UZ Brussel have been screened for STEC since 1987. At first (1987–1990) detection of STEC in feces was done by culture, biochemical methods and the Vero cell Cytotoxicity Assay. Suspicious strains were sent to the Central Public Health Laboratory in London for DNA hybridization with Stx1 and Stx2 probes [10]. In 1991 molecular methods were introduced in our laboratory and until 2007 cultures of fecal samples on MacConkey (MAC) agar were analyzed with conventional PCR for the detection of *stx* genes as described by Karch & Meyer [11;12;15]. Because these primers were not able to detect the *stx2f* subtype, the latter was replaced by culture on sorbitol MAC agar bi-plates with and without cefixime and tellurite ((CT-)SMAC) followed by an in-house multiplex PCR for the combined detection of all *stx1* (1a, 1c and 1d) and *stx2* (2a to 2g) subtypes in 2008 [13;16;17].

It was always attempted to retrieve a STEC isolate from each PCR-positive culture by testing at least 20 colonies for the presence of *stx* genes and all isolated STEC strains were stored at -80 °C. In approximately 30% of the PCR-positive cultures it was not possible to isolate a STEC using this algorithm.

Also the STEC strain serotyping methods evolved over the years. In 1987 O157 and H7 antisera were prepared in-house by immunization of rabbits and these were in use until 2013. Commercially available antisera for the O serogroups O26, O103, O111, O121, O145 (Statens Serum Institut (SSI), Copenhagen, Denmark) were added to our slide-agglutination assay in 1998 and the SSI O157 antiserum replaced our in-house antiserum in 2013. Also in 2013 the sequence based method *gnd*-typing was introduced for molecular O serogrouping of STEC [18]. The motility of the strains was identified with the motility indole urease (MIU) test, but characterization of H flagellar antigen types other than H7 was never performed in-house. Only isolates of which the O serogroup could not be determined in-house were sent to the Laboratory Centre for Disease Control (Ottawa, Canada), to Public Health England (Colindale, England), and to the SSI for complete serotyping over the years.

Information regarding the strains and the age, sex and clinical presentation of the according patients was collected and archived in a database.

### STEC strains included in this study

In order to restrict the analysis to STEC from patients belonging to the UZ Brussel’s catchment area, strains and samples submitted through other laboratories were not taken into account. We solicited our STEC database and it contained data about 626 STEC isolates derived from UZ Brussel patients between 1987 and 2014. Four strains were no longer available in our cryocollection (isolation years: 1991, 1994, 1995 and 2003) and 16 isolates (isolation years: 2 1991, 1 1993, 2 1994, 4 1995, 1 1997, 3 2003, 1 2006, 1 2010, 1 2012) could not be cultivated after prolonged storage at -80 °C. As these isolates were not taken into account, 606 strains were included for further characterization in this study.

### Characterization of STEC strains in this study

#### Virulence genes and *stx* subtyping

Strains were retrieved from the freezer and cultured on (CT-)SMAC. DNA was extracted and subjected to conventional PCR assays for the detection of the STEC virulence genes (*stx*, *eaeA* and *ehxA*), enteroaggregative *E*. *coli* virulence genes (*aaiC* and *aggR*) and *stx*-subtyping as described before [6;13;19;20]. These are the PCR assays that were in place at the Belgian NRC STEC/VTEC for routine characterization of STEC at the time of this study.

#### Serotyping

STEC strains with previously undetermined serotypes were subjected to *gnd*-typing for molecular O serogrouping [18].

#### Molecular typing of O157 isolates by IS629-printing

In order to assess their phylogenetic relatedness, all O157 strains were analyzed with a genotyping method based on the variable presence of insertion sequence 629 (IS629) throughout the O157:H7 genome (IS629-printing). This rapid molecular technique consists of two 16-plex conventional PCR assays targeting different IS629 insertion sites throughout the O157 genome [21]. Two strains belong to the same IS629-type when their fingerprints are indistinguishable. Strains with at least two differing bands are considered unrelated. Strains with only one band different between them are considered related or unrelated dependent on epidemiological and typing data, including the timeframe and geographical area in which the strains were isolated.

#### *Escherichia albertii* detection

Because *stx2f*-positive *Escherichia albertii* have been described to be misclassified as STEC, all *stx2f*-positive strains were tested with a multiplex PCR assay for the detection of *E*. *albertii* housekeeping genes (*clpX*, *lysP* and *mdh*) and screened for the presence of the *cdtB* gene, a common virulence characteristic of *E*. *albertii* [22–24].

### Annual STEC isolation and positivity rates

The annual STEC isolation rate since the introduction of the first PCR assay in 1991 was calculated as follows: [(number of isolated STEC/number of unduplicated fecal samples analyzed)] per year. For this analysis, the 20 lost strains that could not be retrieved from the freezer were included in the number of isolates (*n*_total_ = 626). The detection of all *stx* subtypes was only possible since 2008 when our current culture-PCR assay including the *stx2f* subtype was introduced. The yearly positivity rate of STEC infection in our patient population since 2008 was assessed using following formula: [(number of *stx* PCR-positive cultures (with and without strain isolation)/number of unduplicated fecal samples analyzed)] per year.

### Statistical analysis

Statistical calculations were performed with the Fisher’s exact test and the Pearson’s chi-square test using the Analyse-it v2.26 software (Analyse-it Software Ltd, Leeds, United Kingdom). A p-value of ≤0.05 was considered statistical significant. Cluster analysis of the IS629 fingerprints was performed with the BioNumerics 7.6 software (Applied Maths, Sint-Martens-Latem, Belgium) using the binary band based Dice coefficient and the unweighted pair group method using arithmetic averages (UPGMA) after band matching [25].

### Ethics statement

The strains used in this study were collected and stored in the frame of routine diagnostic culture for bacterial fecal pathogens, without additional testing of these samples. Epidemiological data were collected retrospectively from patient charts and anonymously stored in a database in the frame of Decision No 2119/98/EC of the European Parliament and of the Council, concerning the epidemiological surveillance and control of communicable diseases in the Community, as completed by Decision No 1082/2013/EU. As no additional sampling or information was asked from patients, no formal approval from an ethical committee or informed consents were needed.

## Results

### Study population

The six hundred and six included strains were derived from 604 different patients, 334 (55.3%) females and 270 (44.7%) males. A co-infection of STEC O128ab:H- and STEC O176:H- was found in the fecal sample of a baby boy in 2008. A STEC O153:H19 and a STEC O156:H25 was found in two separate fecal samples of a senior male in 2005 and 2006 respectively. The age of the patients ranged from 1 month to 98 years, the majority were children (≤17) (*n* = 417, 68.9%) and 55.7% (*n* = 337) were 6 years or younger. Sixteen comma seven per cent (16.7%; *n* = 101) belonged to the age group between 18 and 65 years old; 14.4% (*n* = 87) were older than 65.

### Annual isolation rate, positivity rate and evolution

**Fig 1:**
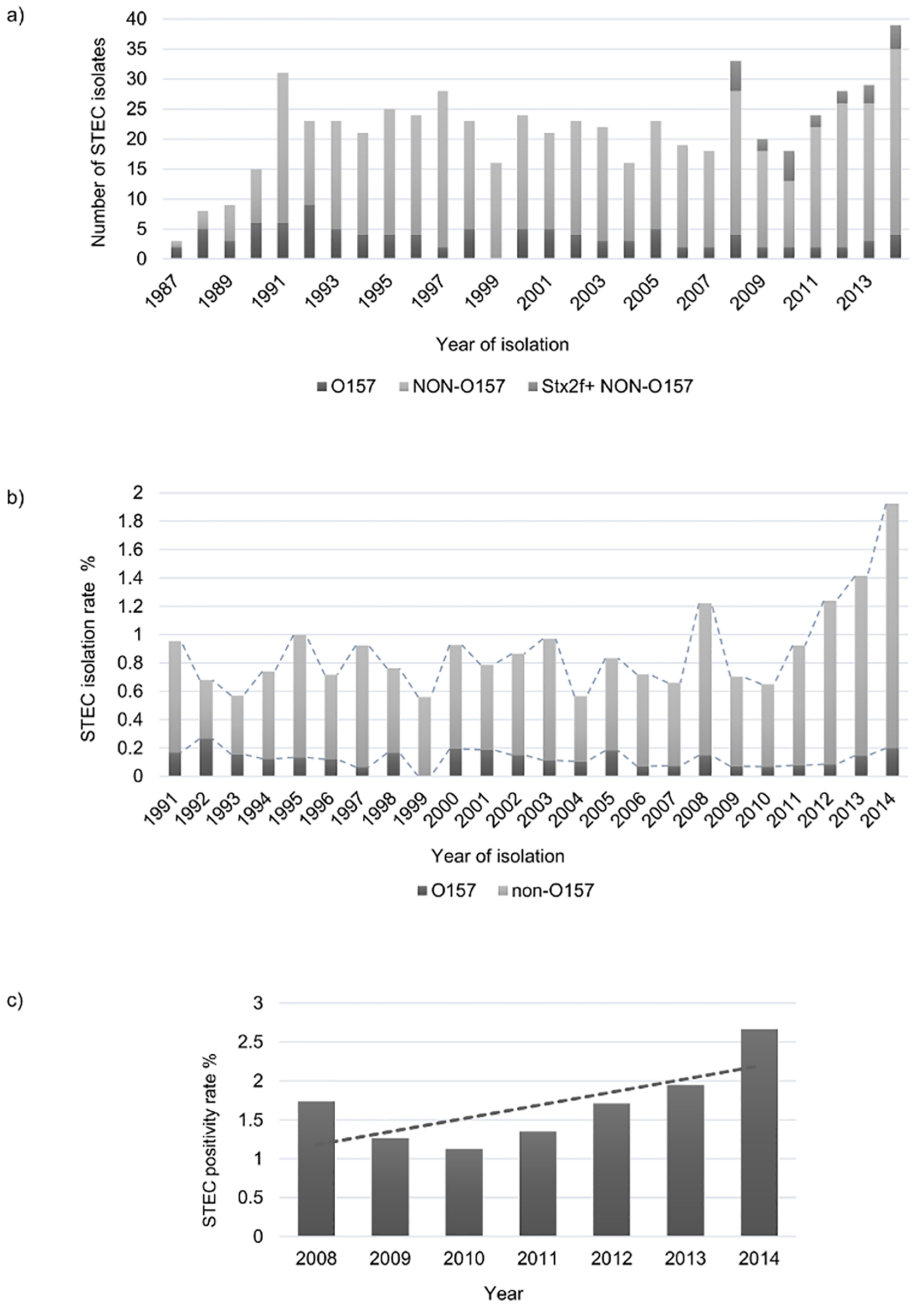
Annual number of STEC isolates (a), annual STEC isolation rate 1991–2014 (b) (---series lines), and annual STEC positivity rate 2008–2014 (c) (---linear trend line).

The first 4 years of the screening (1987–1990), only three, eight, nine and 15 strains were found. Since the introduction of PCR in 1991 a total of 548 STEC were isolated, the *stx2f* positive isolates not taken into account. Since the addition of *stx2f* primers to our assay in 2008 at least two additional STEC were isolated annually, with a total of 23 *stx2f* positive strains over the seven years (2008–2014) (Fig 1a). Overall 83.0% (503/606) of the isolates belonged to non-O157:H7/H- serotypes. Between 3024 and 3564 unduplicated fecal samples were analyzed yearly from 1991 to 1998; from 1999 to 2012 the number varied between 2337 and 2928; and the last 2 years the amount decreased to 2053 (2013) and 2026 (2014). The STEC isolation rate (including the strains that could not be retrieved from the freezer) was the lowest in 1999 (0.6%, 16/2867) and the highest in 2014 (1.9%, 39/2026) (Fig 1b). While the isolation of STEC O157:H7/H- remained stable over the years (average 0.1%, range 0.0–0.3%), an increase could be noticed in the isolation rate of STEC non-O157 serotypes (Fig 1b). The annual positivity rate of STEC infection (culture-PCR *stx*-positive unduplicated stool samples) among our patients since 2008 varied between 1.1% (33/2928) in 2010 and 2.7% (54/2026) in 2014 (Fig 1c).

### STEC serotypes and IS629-printing of STEC O157:H7/H-

Sixty-four strains could not be serotyped (Ounk) with the used methods. The remaining 542 isolates belonged to 72 different O-serovars; O157 (*n* = 103, 17.0%), O26 (*n* = 77, 12.7%), O146 (*n* = 41, 6.8%), O111 (*n* = 38, 6.3%), O103 (*n* = 37, 6.1%), O91 (*n* = 28, 4.3%), O128 (*n* = 20, 3.3%) and O145 (*n* = 20, 3.3%) were the most prevalent. Because the flagellar H-antigen was not typed for all strains, the isolates were divided in seropathotypes based on their O-serogroup only. One hundred and three (19%) strains belonged to seropathotype A (O157:H7/H-), 175 (28.9%; 5 serogroups) isolates to seropathotype B (HUS and outbreak associated) and 162 (26.7%; 19 serogroups) and 102 (16.8%; 47 serogroups) to seropathotype C (HUS associated) and D (diarrhea associated) respectively (Table 1).

**Table 1:** O-serogroups isolated in the present study: Classification in seropathotypes and association with HUS of STEC.

Seropathotype	Serogroup^a^	# Isolates (%, *n*_*total*_ = 606)
**A**: *high incidence*, *commonly involved in outbreaks*, *associated with HUS/HC*	**O157**	103 (17.0)
**B**: *moderate incidence*, *uncommonly involved in outbreaks*, *associated with HUS/HC*	**O26**	77 (12.7)
	**O111**	38 (6.3)
	**O103**	37 (6.1)
	**O145**	20 (3.3)
	O121	3 (0.5)
**C**: *low incidence*, *rarely involved in outbreaks*, *associated with HUS/HC*	O146	41 (6.8)
	O91	28 (4.6)
	O128	20 (3.3)
	O118	18 (3.0)
	O113	14 (2.3)
	**O63**	11 (1.8)
	O55, O117	6 (1.0)
	O115	3 (0.5)
	O5, O80, O101, O104, **O172**, **O178**	2 (0.3)
	O18, **O148**, O177	1 (0.2)
**D**: *low incidence*, *rarely involved in outbreaks*, *not associated with HUS/HC*	O182	15 (2.5)
	O8	5 (0.8)
	O3, O76, O84, O166, O174	4 (0.6)
	O6, O15, O20, O38, O79, O156, O162, O165	3 (0.5)
	O77, O140, O150, O152, O153, O181	2 (0.3)
	O1, O2, O4, O9, O14, O24, O45, O65, O78, O87, O92, O93, O98, O102, O107, O110, O116, O127, O147, O163, O169, O171, O176, O179, O183, O186	1 (0.2)
Unknown	Ounk	64 (10.6)

^a^O-serogroups associated with **HUS** in this study are in bold. The other O-serogroups previously associated with HUS were classified according to Tozzoli and Scheutz [26]

STEC O157 were significantly more present in the age group younger than 18 years old (80/417 vs. 23/188, p = 0.0353), while they were significantly less frequent in patients between 18 and 65 years of age (8/101 vs. 95/504, p = 0.0077).

The STEC O157 showed to be a heterogeneous group as 58 strains had a unique IS629-type (at least one band apart from the others) and the remainder could be subdivided in 17 distinct IS629-clusters each containing 2 to 9 strains with an identical IS629 fingerprint. In eight cases two strains belonging to the same cluster might have had a common source based on their isolation date and genes. However, we were not aware of the occurrence of any outbreak during the studied time frame.

### Shiga toxin subtypes and *Escherichia albertii*

All *stx* subtypes except *stx1d* and *stx2g* were identified (Table 2). The majority of the strains only possessed one *stx* subtype (*n* = 450, 74.3%), 149 (24.6%) and 6 (1.0%) isolates carried two and three subtypes, respectively. One STEC O3:H2 strain isolated in 1995 from a 10-year-old girl with diarrhea even tested positive for four different *stx* subtypes: *stx1a*, *stx2a*, *stx2c* and *stx2d*. The most prevalent subtype was s*tx1a* (*n* = 229, 37.8%) followed by *stx2a* (*n* = 59, 9.7%) and *stx1c+stx2b* (*n* = 54, 8.9%). In 2008 *stx2f* primers were added to our PCR assay and 12% (23/191) of the isolated STEC since that moment carry this subtype. Other significant differences in the occurrence of some *stx* subtypes before and after 2008 include the increase of *stx1c* (1.7% vs. 11.5%, p < 0.0001) and the decrease of *stx2a*+*stx2c* (5.1% vs. 0.5%, p = 0.0047).

**Table 2:** Distribution of the *stx*-subtypes.

Timeframes	A		B		C		D	
	1987–2014 UZ Brussel *n*_*total*_ = 606		1987–2007 UZ Brussel *n*_*total*_ = 415		2008–2014 UZ Brussel *n*_*total*_ = 191		2008–2010 Brussels capital region	
*stx* subtype^a^	n	%	n	%	n	%	*n*_*total*_ = 139	%
***stx1***	**258**	**42.6**	**184**	**44.3**	**74**	**38.7**	**58**	**41.7**
stx1a*	228	37.6	176	42.4	52	27.2	45	32.4
stx1c*	29	4.8	7	1.7	22	11.5	13	9.4
***stx2***	**223**	**36.8**	**145**	**34.9**	**78**	**40.8**	**55**	**39.6**
stx2a**	59	9.7	34	8.2	25	13.1	8	5.8
stx2b*	39	6.4	30	7.2	9	4.7	4	2.9
stx2c**	41	6.8	35	8.4	6	3.1	10	7.2
stx2d*	23	3.8	14	3.4	9	4.7	6	4.3
***stx2e***	7	1.2	3	0.7	4	2.1	4	2.9
stx2f*	23	3.8	0	0.0	23	12.0	18	12.9
*stx2a*, *stx2b*	1	0.2	0	0.0	1	0.5	1	0.7
*stx2a*, stx2c**	22	3.6	21	5.1	1	0.5	3	2.2
*stx2a*, *stx2c*, *stx2d*	1	0.2	1	0.2	0	0.0	0	0.0
*stx2a*, *stx2d*	4	0.7	4	1.0	0	0.0	0	0.0
*stx2b*, *stx2d*	1	0.2	1	0.2	0	0.0	0	0.0
*stx2c*, *stx2d*	2	0.3	2	0.5	0	0.0	1	0.7
***stx1+stx2***	**125**	**20.6**	**86**	**20.7**	**39**	**20.4**	**26**	**18.7**
***stx1a*, *stx2a***	9	1.5	6	1.4	3	1.6	5	3.6
*stx1a*, *stx2a*, *stx2c*	2	0.3	1	0.2	1	0.5	1	0.7
*stx1a*, *stx2a*, *stx2c*, *stx2d*	1	0.2	1	0.2	0	0.0	0	0.0
*stx1a*, *stx2a*, *stx2d*	2	0.3	2	0.5	0	0.0	0	0.0
*stx1a*, stx2b**	25	4.1	18	4.3	7	3.7	2	1.4
*stx1a*, stx2c**	31	5.1	25	6.0	6	3.1	13	9.4
*stx1a*, *stx2d*	1	0.2	1	0.2	0	0.0	0	0.0
*stx1c*, stx2b*	54	8.9	33	8.0	21	11.0	5	3.6
*stx1c*, *stx2b*, *stx2d*	1	0.2	0	0.0	1	0.5	0	0.0

Columns A, B, C: *stx*-subtypes detected during different time frames of this study; column D: *stx*-subtypes detected in a previous study conducted in the Brussels capital region between 2008–2010 [13].

^a^*stx* subtypes associated with **bloody diarrhea (BD)** are in bold, with HUS are underlined, and with **HUS and BD** in this study are in bold and underlined.

*: significantly more prevalent in non-O157 serotypes.

**: significantly more prevalent in STEC O157:H7/H-.

Subtypes *stx1a* (228/503 vs. 1/103, p < 0.0001), *stx1c* (29/503 vs. 0/103, p = 0.0078), *stx2b* (39/503 vs. 0/103, p = 0.0011), *stx2d* (23/503 vs. 0/103, p = 0.0252), *stx2f* (23/503 vs. 0/103, p = 0.0252), *stx1a+stx2b* (25/503 vs. 0/103, p = 0.0171), and *stx1c+stx2b* (54/503 vs. 0/103, p < 0.0001) were more present in non-O157 strains in comparison to O157 isolates; while *stx2a* (19/103 vs. 40/503, p = 0.0011), *stx2c* (30/103 vs. 11/503, p < 0.0001), *stx1a+stx2c* (30/103 vs. 0/503, p < 0.0001) and *stx2a+stx2c* (21/103 vs. 1/503, p < 0.0001) were more frequent in STEC O157.

*Stx1c*, *stx2b*, *stx2d*, *stx2e*, *stx1a+stx2b*, *stx1a+stx2c* and *stx1c+stx2b* were found in patients suffering from bloody diarrhea. *Stx1a*, *stx2a*, *stx2c*, *stx2a+stx2c* and *stx1a+stx2c* were detected in patients diagnosed with HUS and bloody diarrhea.

Out of the 23 *stx2f* positive strains, one was associated with HUS. The *clpX*, *lysP*, *mdh* and *cdtB* genes were detected in one of the *stx2f* positive strains indicating this isolate belonged to the *E*. *albertii* species. The bacterium was isolated in 2013 from a 38-year-old female oncology patient who suffered from diarrhea. The strain also carried the *eaeA* gene and could not be serotyped with agglutination or *gnd*-typing.

### Virulence genes

All STEC O157 and many of the non-O157 strains (229/503, 45.5%) were ‘typical’ EHEC carrying both the *eaeA* and the *ehxA* genes. Only *ehxA* or *eaeA* were found in 24.3% (122/503) and 7.2% (36/503) of the non-O157 isolates, respectively (Table 3). One *stx2a* positive STEC of the O104:H4 serotype carried the *aaiC* and *aggR* genes typical for enteroaggregative *E*. *coli*. One hundred and fifteen strains (19.0%) did not test positive for any of the virulence genes. Since 2008 a higher percentage of ‘atypical’ STEC carrying only *eaeA* or *ehxA* were isolated in comparison with the years before. Twenty-two out of the 23 only *eaeA* positive STEC isolated since 2008 carried the *stx2f* subtype.

**Table 3:** Distribution of the detected virulence genes.

Timeframes	A		B		C	
	1987–2014 UZ Brussel *n*_*total*_ = 606		1987–2007 UZ Brussel *n*_*total*_ = 415		2008–2014 UZ Brussel *n*_*total*_ = 191	
Virulence genes	n	%	n	%	n	%
***eaeA***	**368**	**60.7**	**266**	**64.1**	**102**	**53.4**
*eaeA*	36	5.9	13	3.1	23	12.0
*eaeA*, *ehxA*	332	54.8	253	61.0	79	41.4
***ehxA***	**454**	**74.9**	**319**	**76.9**	**135**	**70.7**
*ehxA*	122	20.1	66	15.9	56	29.3
*eaeA*, *ehxA*	332	54.8	253	61.0	79	41.4
***aaiC*, *aggR***	**1**	**0.2**	**0**	**0**	**1**	**0.5**
***None of the above***	**115**	**19**	**83**	**20.0**	**32**	**16.8**

Columns A, B, C: virulence genes detected during different time frames of this study

### Clinical manifestations and characteristics correlated to HUS

Clinical information was available for 531 patients. Uncomplicated diarrhea was the most common gastrointestinal manifestation (*n* = 275, 51.9%) followed by bloody diarrhea (*n* = 72, 13.6%) and abdominal pain (*n* = 68, 12.8%). Nonspecific symptoms were noted in 17.5%. Twenty-three STEC strains (4.3%) were isolated from the stool samples of patients suffering from HUS (Table 4). A similar percentage of the female (13/334, 3.9%) and male (10/270, 3.7%) patients from whom a STEC was isolated were affected with HUS. No statistical significant correlations between HUS and age were identified. The majority of the HUS-related strains were typical *eaeA* and *ehxA* positive EHEC (*n* = 19) belonging to seropathotypes A or B. One strain had the O63:H6 serotype and was *eaeA* and *stx2f* positive (Table 3). The O157:H7/H- serotype (14/23 vs. 77/510), the *stx2* subtype in general (22/23 vs. 285/510) and *stx2a* (18/23 vs. 73/510) more specifically, as well as strains harbouring the virulence genes *eaeA* (22/23 vs. 295/510, p = 0.0002) and *ehxA* (22/23 vs. 377/510, p = 0.0199) were significantly more frequently isolated from patients suffering from HUS (all p < 0.0001).

**Table 4:** Characteristics of the 23 strains isolated from HUS patients.

Strain	Serotype	*stx* subtype	*eaeA*	*ehxA*	Sex	Age category	≤6 years	Isolation year
**EH10**	O172:H-	*stx2a*	Y	Y	M	≤17	Y	1988
**EH88**	O157:H7	*stx2a*, *stx2c*	Y	Y	F	≤17	Y	1991
**EH118**	O157:H7	*stx2a*, *stx2c*	Y	Y	F	≤17	Y	1992
**EH315**	O145:H-	*stx2a*	Y	Y	M	≤17	Y	1996
**EH345**	O157:H7	*stx2a*	Y	Y	M	≤17	Y	1996
**EH346**	O157:H7	*stx2a*	Y	Y	F	≤17	Y	1996
**EH403**	O157:H-	*stx2a*	Y	Y	F	≤17	Y	1997
**EH502**	O157:H7	*stx2a*, *stx2c*	Y	Y	F	>65	N	1998
**EH526**	O157:H7	*stx2a*, *stx2c*	Y	Y	F	≤17	N	1998
**EH839**	O178:H19	*stx2a*	N	Y	M	>65	N	2001
**EH1060**	O157:H7	*stx2a*, *stx2c*	Y	Y	F	≤17	Y	2002
**EH1061**	O157:H7	*stx2c*	Y	Y	F	≤17	Y	2002
**EH1241**	O157:H-	*stx2c*	Y	Y	M	≤17	Y	2003
**EH1283**	O157:H7	*stx2a*, *stx2c*	Y	Y	M	≤17	Y	2004
**EH1382**	O157:H7	*stx2a*	Y	Y	F	>65	N	2005
**EH1412**	O157:H7	*stx2a*, *stx2c*	Y	Y	M	≤17	Y	2005
**EH1811**	O111:H8	*stx1a*, *stx2a*	Y	Y	F	18–65	N	2009
**EH1960**	O157:H7	*stx2a*	Y	Y	M	≤17	Y	2010
**EH2123**	O63:H6	*stx2f*	Y	N	F	>65	N	2012
**EH2149**	O26:Hunk	*stx2a*	Y	Y	F	≤17	Y	2012
**EH2222**	O148:H8	*stx2c*	Y	Y	M	≤17	N	2012
**EH2298**	O145:Hunk	*stx2a*	Y	Y	M	≤17	Y	2013
**EH2300**	O103:Hunk	*stx1a*	Y	Y	F	≤17	N	2013

H- = strain is not motile; Hunk = unknown H antigen; Y = yes; N = no; F = female; M = male

## Discussion

This study distinguishes itself from other studies because all samples that were submitted for aerobic stool culture to our laboratory since 1987 were screened for the presence of STEC (non-O157 as well as O157). Laboratories often only screen for STEC when it is specifically requested by the physician or implement an algorithm based on the clinical symptoms or the appearance of the specimen to decide whether or not to screen. As a result, most studies that describe human STEC infections focus on a specific group of STEC or patients or do not encompass such a long period of time [13;27–29].

Since the introduction of culture-PCR in 1991 the average STEC isolation rate among our unselected patient stool specimens was 0.9% (range 0.6%–1.9%). The average positivity rate of *stx*-positive stool samples between 2008 and 2014 was 1.7%. Results of a recent study we conducted indicate that this number would probably even be higher if a culture-independent real-time molecular detection method was applied instead of our in-house *stx* PCR applied after culture [30]. Using this culture-based method implies that all STEC that cannot grow on SMAC(-CT) agar will not be detected. Furthermore, culturing STEC can cause the loss of the *stx* phage which could also result in STEC being missed by our routine methods [31].

In 2014 Foodnet noted the incidence of STEC O157 and non-O157 in the United States of America to be 0.91 and 1.43 per 100000 inhabitants, respectively [32]. According to the European Centre for Disease Prevention and Control (ECDC) the notification rate of STEC infections between 2010 and 2012 was 1.7 cases/100000 [4]. However, these notification data probably underestimate the true incidence of STEC infection and a recent study conducted in Germany estimated the true annual incidence of STEC-associated gastrointestinal infections at 35/100000 [33]. Even though substantial efforts have already been made to prevent products contaminated with STEC from entering the food chain, the burden on public health remains high. More efficient measures are needed to control the carriage of STEC in animals and its spread to the environment on a large scale. A multipronged approach will be needed to reduce the burden of STEC infections in the future [34].

Approximately one third (30.2%) of the affected patients in this study did not suffer from diarrhea or HUS, of which 17.5% did not even show any gastrointestinal symptoms at all. The proportion of HUS among the isolated STEC was 4.3%, which is lower than the 8–12% among the notified STEC infections in Europe [4]. This is not unexpected as in this study all fecal samples were screened for STEC, regardless of the clinical symptoms of the patients, while international screening guidelines suggest to only screen the samples of patients suffering from diarrhea and/or HUS [35]. The importance of this asymptomatic carriage should not be underestimated, as a seemingly innocent STEC strain can cause severe disease in another host, as seen in outbreaks.

As already described in previous conducted studies the presence of the O157:H7/H- serotype, the *stx2* subtype in general, *stx2a* more specifically and the virulence genes *eaeA* and *ehxA* were more frequent in HUS patients [28;36;37]. Nevertheless, the STEC strains isolated from HUS patients here were quite heterogeneous and some of them carried LPS antigens and/or *stx* subtypes not commonly associated with severe disease outcome. The most peculiar HUS-STEC was an *stx2f+*, *eaeA+*, *ehxA-* STEC O63:H6/H- retrieved from a 87-year-old female patient who presented at the emergency room with complaints of diarrhea, vomiting and abdominal pain. Further analyses revealed she was developing HUS and she was treated during a hospitalization for over 20 days.

The incidence of STEC O157 remained relatively stable, which was also seen in a study encompassing the same period in England and Wales [27]. An increasing trend in the isolation rate of non-O157 serotypes was observed since 2011 with a peak in 2014, but further surveillance will need to reveal if the high incidence seen in 2014 was an outlier or the start of a true raise.

Recently the characteristics of all human STEC isolated in Germany between in 1997 and 2013 were published by the Robert Koch Institute [38]. Some parallels could be noticed between our results and the German nationwide statistics. A very diverse group of serotypes was detected, but about half of the isolates are made up by only four or five O serogroups. Besides O157 and O26 the top four in Germany is completed with O103 and O91. In our hospital O146 and O111 fulfill the top four while O103 and O91 rank fifth and sixth, respectively. Obviously O104:H4 was the major serovar detected in Germany in 2011, but none of our patients were part of this large outbreak. One Agg-STEC O104:H4 was isolated one year later in the bloody stool sample of a female returning from Tunisia; PFGE typing showed the isolate was closely related but not identical to the German outbreak strain [39].

In Europe a significant increasing trend in the number of cases was observed for serotypes O26:H11, O145:H- and O63:H6 during 2008–2012 [4]. In our hospital the first detection of the O63:H6 serotype coincided with the addition of *stx2f* primers to our PCR assay in 2008. All O63:H6 strains in this study were positive for the *stx2f* subtype, providing an explanation for its sudden occurrence. This Stx subtype is genetically and immunologically the most distant and is often not detected by commercial assays [30;40–42]. Since 2008 12% of all our STEC isolates were *stx2f* positive which is comparable to the 13% that was seen in a study conducted in the Brussels-Capital region and the 16% that was noted in our neighboring country the Netherlands [13;43]. Deeper analysis revealed one of the *eaeA* and *stx2f* positive strains belonged to the *E*. *albertii* species. This species is closely related to the *E*. *coli* species and because they possess the *eaeA* gene, sometimes in combination with the *stx2f* gene, they are often misclassified as EPEC or STEC. Even though the clinical significance of *E*. *albertii* still needs to be fully defined, sporadic human infections and even association with an outbreak have been described [22;44;45]. To the best of our knowledge this is the first report of an *E*. *albertii* that possesses the *stx2f* gene isolated from a Belgian patient.

From 2008 to 2010 a study was conducted in the Brussels-Capital region to assess the incidence and virulence determinants of STEC infections [13]. When comparing the prevalence of *stx*-subtypes detected in that study with those found in the present study from 2008 to 2014 a few differences can be noted (Table 2). A raise was seen for *stx2a* (13.1% vs. 5.8%, p = 0.0283) and *stx1c + stx2b* (11% vs 3.6%, p = 0.0138). In both cases the majority of strains were isolated since 2011 (22/25 for *stx2a* and 17/21 *for stx1c+stx2b*). The *stx2a*-positive isolates belonged to 12 different O-serovars, while the majority (14/17) of the *stx1+stx2b*-positives were restricted to the O-serogroups O146 and O128.

Our collection of strains showed a high heterogeneity. Several rather rare serotypes were associated with HUS and as the list of serotypes belonging to seropathotype C keeps growing, the whole concept of the classification in seropathotypes is being challenged [46;47]. Here we analyzed a limited number of virulence genes, but other researchers studying extensive genetic data were also not able to define a clear correlation between gene content and disease outcome [48–50]. This underlines the importance of among other things the host characteristics in the severity of the disease.

## Conclusions

The annual positivity rates of STEC infection ranged from 1.1% to 2.7%, with an average positivity rate of 1.7%. The isolation rate of STEC O157:H7/H- remained stable over the years while an increase was noticed in the isolation rate of non-O157 serotypes. A high genetic diversity was seen among the strains, indicating that STEC infections in our hospital are mostly sporadic. Only 4.3% of the patients suffered from HUS, while approximately one third of the STEC carriers were asymptomatic. We have described the first Belgian STEC *stx2f* HUS case and *stx2f* positive *E*. *albertii* infection. As recently seen in our neighbouring country the Netherlands human STEC *stx2f* infections were more common in our hospital than generally expected.

## Supporting information

S1 Table Overview of the STEC strains and data included in this study. (XLSX)
